# New insights into *SRY *regulation through identification of 5' conserved sequences

**DOI:** 10.1186/1471-2199-9-85

**Published:** 2008-10-14

**Authors:** Diana GF Ross, Josephine Bowles, Peter Koopman, Sigrid Lehnert

**Affiliations:** 1 Division of Molecular Genetics and Development, Institute for Molecular Bioscience, The University of Queensland, Brisbane 4072, Australia; 2 Commonwealth Scientific and Industrial Research Organisation (CSIRO), Division of Livestock Industries, Queensland Bioscience Precinct, The University of Queensland, Brisbane 4072, Australia

## Abstract

**Background:**

*SRY *is the pivotal gene initiating male sex determination in most mammals, but how its expression is regulated is still not understood. In this study we derived novel *SRY *5' flanking genomic sequence data from bovine and caprine genomic BAC clones.

**Results:**

We identified four intervals of high homology upstream of *SRY *by comparison of human, bovine, pig, goat and mouse genomic sequences. These conserved regions contain putative binding sites for a large number of known transcription factor families, including several that have been implicated previously in sex determination and early gonadal development.

**Conclusion:**

Our results reveal potentially important *SRY *regulatory elements, mutations in which might underlie cases of idiopathic human XY sex reversal.

## Background

Sex in mammals normally correlates with the presence or absence of the Y chromosome. Male sex determination in almost all mammals is directly caused by the correct expression and function of a single Y-linked gene, *SRY*[1-4]. *SRY *activity in males causes the bipotential gonad, the genital ridge, to set off on the path to becoming a testis. If the fetal genital ridge does not express *SRY*, ovary development is initiated instead. A majority of gonadal dysgenesis cases cannot be attributed to mutations within or immediately 5' of *SRY*, or to any other gene known to have a role in sex determination. We hypothesise that this is because *SRY's *regulatory regions are uncharted, therefore providing no means to check specific areas for mutation.

*SRY *carries out a similar function in all mammals in which it is present, but displays a high degree of variability between species. This situation is thought to result from the location of *SRY *on the Y chromosome, exposing it to a higher rate of mutation compared to autosomal genes, thereby leading to DNA degradation and even loss [5]. The region of *SRY *best conserved between species is the high mobility group (HMG) box, which confers the encoded protein its transcription factor role by allowing it to bind and bend DNA [6,7]. Outside the HMG box, *SRY *is very poorly conserved between species. This lack of conservation has made it difficult to define functional motifs required for the role of SRY protein in directing male sex determination.

The regulation of *SRY *is under tight control to ensure its expression at the right time, place and level necessary to initiate male sex determination. In mice, delayed onset of *Sry *expression, or reduced levels of *Sry *expression, is known to cause full or partial XY sex reversal [8-10]. Therefore, an understanding of how *SRY *expression is regulated is an important part of the overall picture of its functions in male sex determination and of how disturbances in function can lead to disorders of sex development.

As with the *SRY *coding region, sequences beyond the transcription unit of *SRY *are very poorly conserved between species, a situation that has contributed to an almost total lack of understanding of how the expression of this gene is regulated. Comparative genomics is normally a powerful tool for identifying biologically important gene regulatory regions, based on the conservation of functional regulatory modules being under selective pressure during evolution [11-13], but this method has shown only limited success in studies of *SRY *to date. Although mice are most useful for a range of developmental and functional genetic studies, their utility in comparative genomics is limited by their unusually high rate of sequence drift, thought to be linked to their short generation time [14].

Progress in identifying potential gene regulatory motifs through comparative genomics relies on the availability of genome sequences from a range of non-murine mammals. A study analysing non-coding sequences in 39 bovine, human and mouse gene orthologues revealed 73 putative regulatory intervals conserved between bovine and human genes, only 13 of which were also conserved in mice [15]. Further comparative genomic analysis of these regions showed that the homology to human is highest in bovine, and weakest in the mouse. Other studies also point to an excellent conservation of bovine and human sequences in the promoter region of genes such as *Oct4*, but relatively poor conservation of the corresponding mouse sequences [16].

In the present study we generated novel bovine and caprine *SRY *5' sequence data in order to conduct comparative genomic analysis of 5' sequences from human, bull, pig, goat and mouse *Sry*. In this way we identified four novel sequence intervals that may be important for the correct regulation of *SRY *expression and therefore for correct function of SRY in mammalian sex determination. The identification of these candidate regulatory regions provides a focus for efforts to discover new mutations associated with human idiopathic XY sex reversal.

## Results

### Generation of novel Sry genomic flanking sequence from bovine and caprine BACs

In order to provide new tools for comparative genomic analysis of potential *SRY *5' regulatory sequences, we first generated novel flanking sequence from the bovine and caprine *SRY *genes. The BAC clone RP42-95D10 containing bovine *SRY *[17] was found by Southern blotting and polymerase chain reaction (PCR) to contain a 15 kb *Eco*R1 fragment harbouring *SRY *(data not shown). This fragment was subcloned, and sequenced to five times coverage [GenBank EU581861].

Alignment of the bovine sequence with published human [EMBL: NT_011896.9 nucleotides 5177–21272] and mouse [EMBL: NT_078925.6 nucleotides 1917040–1934040] SRY 5' sequence allowed the preliminary identification of several potentially conserved sequence blocks. We generated corresponding fragments of the goat *SRY *5' region by PCR using as template a goat BAC clone containing *SRY *and known to cause female to male sex reversal in mice [18]. These fragments were sequenced, aligned, and appended to existing goat *SRY *sequence where possible, and used for further analysis [Genbank EU581862, EU581863, and EU581864].

### Comparative genomic sequence analysis

**Figure 1:**
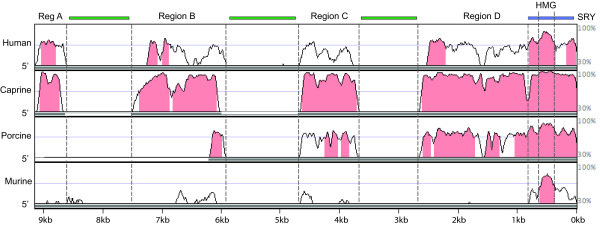
**Homology of human, caprine, porcine and mouse *SRY *5' sequences to bovine SRY**. Pink shading indicates 70% or higher homologies calculated over 100 bp. Peaks of homology are labelled Region A to D above the graph. Repetitive elements (LINEs and SINEs) are indicated in green, and the *SRY *coding region in blue. Grey line below each graph shows the extent of sequence used.

**Figure 2:**
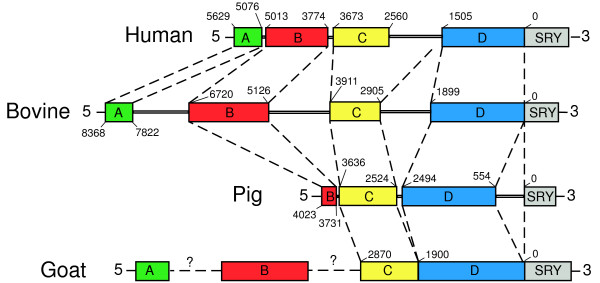
**Spacing of and co-ordinates of conserved *SRY *5' regions in different species**. Sequence information for porcine region A was not available for this study. Numbering represents number of nucleotides 5' to the transcription start site in each species, ? denotes unknown positions.

**Figure 3:**
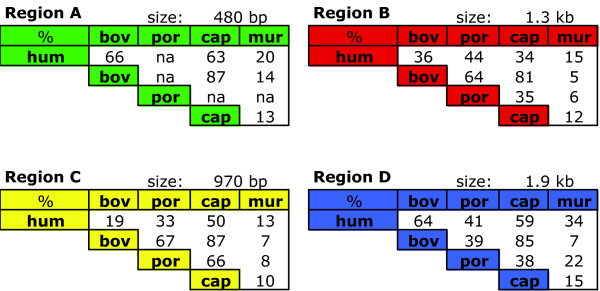
**DNA sequence homologies calculated across the whole of regions A, B, C and D**. Species are human (hum), bovine (bov), porcine (por), caprine (cap), and murine (mur). na, porcine sequence not available.

We next used the novel 15 kb of bovine *SRY *5' sequence as a reference point for comparative genomic studies. VISTA alignment of the bovine sequence with human, porcine (4.6 kb) [19], caprine (individual regions described above), and mouse (17 kb), revealed four sequence blocks of significant homology (Figure 1). These blocks (A, B, C and D) from human, caprine and porcine *SRY *displayed at least 50% nucleotide identity to bovine sequence by VISTA analysis using 100 bp windows. The four conserved blocks were separated by non-conserved sequence, the length of which varied between species (Figure 2). In the goat no intervening sequences were detected between region C and D. The main features of each conserved block are as follows:

Region A (480 bp) lies about 8.3 kb upstream of the start of transcription in bovine *SRY *(5.6 kb in human; Figure 2). It showed more than 70% conservation in 100 bp windows between bovine, human and caprine sequence over a large proportion of its length using VISTA (Figure 1, pink shading). ClustalW showed overall homology between the three species as 63 – 87% (Figure 3).

Region B (1.5 kb) begins 6.7 kb 5' of the bovine *SRY *start of transcription (5 kb in human; Figure 2). Bovine/human homology, in 100 bp windows of this region, was above 70%, limited to two short sequence intervals (Figure 1). This high homology between bovine and caprine, and moderate homology between bovine and human sequences, was reflected in overall ClustalW homology analysis of these regions (Figure 3). As in region A, homology of mouse sequence was minimal in this region. The available 4.6 kb of porcine genomic sequence stopped partway through this region, but aligned well with bovine sequence (Figure 1).

Region C (1 kb) was found 3.9 kb upstream on the bovine sequence (3.6 kb in human; Figure 2). This was the least conserved area between bovine and human, not reaching 70% in any 100 bp window using the VISTA browser (Figure 1), and only 19% overall by ClustalW (Figure 3). Caprine sequence showed high homology to bovine in this region, porcine intermediate, and mouse negligible (Figures 1, 3).

Region D was found immediately upstream of bovine, human and caprine *SRY*, and so represents the proximal promoter region in these species (1.9, 1.5 and 1.9 kb respectively). This region showed strong to moderate conservation across all species except mouse (Figure 1, 3). Conservation between bovine and human sequences was stronger in this region than other regions (Figure 3).

No additional regions of homology were detected distal to region A within the 15 kb of bovine sequence used as anchor, when compared with 17 kb of human and 16 kb of mouse sequence.

### Conserved transcription factor binding sites

**Figure 4:**
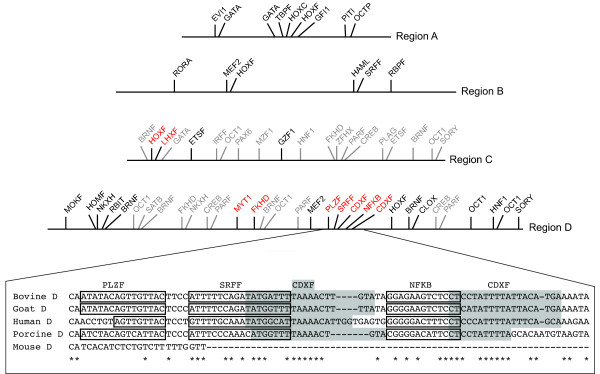
**Conserved transcription factor binding sites in each region of homology**. Black text indicates conservation between 3 species of which one is human, grey text indicates 3-species conservation without human, and red text indicates conservation between 4 species (human, bovine, porcine and caprine). An example of the highly conserved area of region D is shown as a sequence alignment with conserved transcription factor binding sites boxed or shaded.

We next searched for potential transcription factor binding sites in conserved regions A-D in order to evaluate the possible significance of these regions for *SRY *regulation. *In silico *DiAlignTF analysis revealed 210 conserved, canonical transcription factor binding sites across the four regions, representing 38 transcription factor families (Table 1 and 2, Figure 4 and additional file 1). None of the transcription factor binding sites were shown as conserved in the mouse using DiAlignTF, although some nucleotide conservation was detectable when viewed by eye (Additional file 1). To allow us to add levels of significance to the putative sites they were grouped according to their occurrence patterns in the sequences (Table 1): most frequent (total number of times represented in the four regions), most common (number of regions containing each type of site) and level of conservation (number of species containing the site) among the four species examined other than mice. In addition, the matrix similarity score for each site (that is, the similarity of each putative site to the canonical binding site for the relevant transcription factor) is shown in Table 3, as further indication of the likely relevance of each putative binding site.

**Table 1:** Transcription factor binding sites found in *SRY *5' regions

**Family**	**Reg. A**	**Reg. B**	**Reg. C**	**Reg. D**	**TOTAL**
BRNF			b/g/p (×2)	b/g/h (×2) b/g/p (×2)	18

OCT1			b/g/p (×2)	b/g/p (×2) b/g/h (×2)	18

HOXF	b/g/h	b/g/h	**b/g/p/h**	b/g/h	13

PARF			b/g/p	b/g/p (×3)	12

FKHD			b/g/p	b/g/p **b/g/h/p**	10

GATA	b/g/h (×2)		b/g/p		9

CREB			b/g/p	b/g/p (×2)	9

CDXF				**b/g/h/p **(×2)	8

SRFF		b/g/h		**b/g/h/p**	7

MEF2		b/g/h		g/h/p	6

ETSF			b/g/h, b/g/p		6

HNF1			b/g/p	b/g/h	6

SORY			b/g/p	g/h/p	6

NKXH				b/g/h b/g/p	6

LHXF			**b/g/p/h**		4

MYT1				**b/g/h/p**	4

PLZF				**b/g/h/p**	4

NFKB				**b/g/h/p**	4

EVI1	b/g/h				3

TBPF	b/g/h				3

HOXC	b/g/h				3

GFI1	b/g/h				3

PITI	b/g/h				3

OCTP	b/g/h				3

RORA		b/g/h			3

HAML		b/g/h			3

RBPF		b/g/h			3

IRFF			b/g/p		3

PAX6			b/g/p		3

MZF1			b/g/p		3

GZF1			b/g/h		3

ZFHX			b/g/p		3

PLAG			b/g/p		3

MOKF				b/g/h	3

HOMF				b/g/h	3

RBIT				b/g/h	3

SATB				b/g/p	3

CLOX				b/g/h	3

**TOTAL**	27	18	62	103	210

Only sites conserved between 3 or more species are shown. Sites conserved between 4 species are marked in bold. Numbers in parentheses indicate the number of times each binding site was found in the same region. Data are sorted in order of most common to least common transcription factor binding sites. Bovine (b), goat (g), human (h), pig (p), mouse (m).

**Table 2:** Transcription factor family members

**Family**	**Transcription factors**
BRNF	Brn POU domain factors
	BRN2/3/4/5

OCT1	Octamer binding protein
	OCT1/2/3

HOXF	Factors with moderate activity to homeodomain consensus sequence
	Barx2, CRX, GSC, Gsh-1/2, HOX1, HOXA9, HOXB9, HOXC13, NANOG, OTX2, PCE1, PHOX2a/2b, PTX1 pituitary homeobox.

PARF	PAR/bZIP family
	DBP Albumin D-box binding protein, HLF hepatic leukemia factor, TEF Thyrotrophic embryonic factor, VBP PAR-type chicken vitellogenin promoter binding protein.

FKHD	Fork head domain factors
	FHXA/B, FKHRL1 (FOXO), FREAC2/3/4/7 fork head related activators (FOXF2, FOXC1, FOXD1, FOXL1), HFH1/2/3/8 (FOXQ1, FOXD3, FOXI1, Freac-6. FXF1), HNF3B (FOXA2), IlF1 (FOXK2), XFD1/2/3.

GATA	GATA binding factors
	GATA, GATA1/2/3.

CREB	Camp-responsive element binding proteins
	ATF, ATF2/6 activation transcription factors, c-Jun/ATF2 heterodimers, CREB, CREB1/2, CREB2/cJun, E4BP4, TAX/CREB complex, XBP1 X-box-binding protein.

CDXF	Vertebrate caudal related homeodomain Protein
	CDX1/2 Intestine specific homeodomain factor and mammalian caudal related intestinal TF.

SRFF	Serum response element binding factor
	SRF.01/02/03

MEF2	Myocyte-specific enhancer binding Factor
	MEF2, RSRFC4 related to serum response factor, SL1 member of RSRF

ETSF	Human and murine ETS1 factors
	c-Ets-1/2(p54), ELF-2(NERF1a), ELK1, FLI, GABP GA binding protein, GABPB1 GA repeat binding protein beta 1, NRF2 nuclear respiratory factor 2, PDEF Prostate-derived Ets factor, PEA3 polyomavirus enhancer A binding protein 3, ETV4, PU1, SPI1, SpiB.

HNF1	Hepatic nuclear factor 1
	HNF1

SORY	SOX/SRY-sex/testis determining and related HMG box factors
	HBP1, HMGA1/2, HMGIY, SOX5/9, SRY.

NKXH	NKX homeodomain factors
	Hmx2/Nkx5-2 homeodomain transcription factor, NKX31 prostate-specific homeodomain protein, TTF1 thyroid transcription factor

LHXF	Lim homeodomain factors
	LHX3 and LMXB1

MYT1	MYT1 C2HC zinc finger protein
	MyT1 myelin transcription factor, and MyT1L.

PLZF	C2H2 zinc finger protein
	PLZF promyelocytic leukemia zinc finger (TF with 9 Kruppel-like zinc fingers)

NFKB	Nuclear factor kappa B/c-rel
	NF-kappaB (p50 and p65), HIVEP1; ZAS Domain TF human immunodeficiency virus type 1 enhancer-binding protein-1 (HIVEP1), major histocompatibility complex-binding protein-1 (MBP-1), positive regulatory domain II-binding factor (PRDII-BF1)

EVI1	Myleoid transforming protein
	EVI1 ecotropic viral integration site 1 encoded factor, amino-terminal zinc finger domain. MEL1 (MDS1/EVI1-like gene 1) DNA-binding domain 1.

TBPF	TATA-binding protein factors
	ATATA avian C-type LTR TAT box, LTATA Lentivirus LTR TAT box, MTATA muscle TATA box, TATA cellular and viral TATA box elements, and Mammalian C-type LTR TATA box.

HOXC	HOX – PBX complexes
	HOX/PBX binding sites, PBX1, PBX-HOXA9 binding site.

GFI1	Growth factor independence transcriptional Repressor
	GFI1.01/02, GFI1B.01.

PITI	GHF-1 pituitary specific pou domain TF
	Pit1, GHF1.

OCTP	OCT1 binding factor (POU-specific domain)
	OCT1P Octamer-binding factor 1, POU-specific domain)

RORA	v-ERB and RAR-related orphan receptor alpha
	REV-ERBA orphan nuclear receptor rev-erb alpha (NR1D1), RORA/RORA1/2 RAR-related orphan receptor alpha/1/2, RORGAMMA RAR-related orphan receptor gamma, VERBA viral homolog of thyroid hormone receptor alpha1

HAML	Human acute myelogenous leukemia factors
	AML1/CBFA2 Runt domain binding site, AML3 runt-related transcription factor 2/CBFA1

RBPF	RBPJ kappa
	Mammalian transcriptional repressor RBP-Jkappa/CBF1

IRFF	Interferon regulatory factors
	IRF1/2/3/4(NF-EM5, PIP, LSIRF, ICSAT)/7, ISRE interferone stimulated response element.

PAX6	PAX-4/PAX-6 paired domain binding sites
	PAX4 and PAX6 paired domain binding site

MZF1	Myeloid zinc finger 1 factors
	MZF1

GZF1	GDNF-inducible zinc finger gene 1
	GZF1 (ZNF336)

ZFHX	Two-handed zinc finger homeodomain transcription factors
	AREB6 (Atp1a1 regulatory element binding factor 6), deltaEF1 (Delta-crystallin enhancer binding factor, transcription factor 8, zinc finger homeobox 1a), SIP1 (Smad-interacting protein)

PLAG	Pleomorphic adenoma gene
	(PLAG) 1, a developmentally regulated C2H2 zinc finger protein

MOKF	Mouse Kruppel like factors
	MOK2.01/02 Ribonucleoprotein associated zinc finger protein MOK-2

HOMF	Homeodomain transcription factors
	DLX1/2/5, Distal-less 3, EN1 homeobox protein engrailed, HHEX, MSX1/2, NOBOX, S8.

RBIT	Regulator of B-Cell IgH transcription
	Bright, B cell regulator of IgH transcription

SATB	Special AT-rich sequence binding
	Protein SATB1

CLOX	CLOX and CLOX homology (CDP) factors
	CDP cut-like homeodomain protein, transcriptional repressor CDP, CDPCR3, CDPCR3HD, CLOX, CUT2.

List of transcription factor families found in Regions A-D and the specific transcription factors that comprise them.

**Table 3:** Matrix similarity scores for putative binding sites

**Region**	**Site**	**Bovine**	**Human**	**Goat**	**Porcine**	**Mean**
A	HOXF	0.96	0.988	0.947	-	0.965
	
	GATA (a)	0.924	0.963	0.956	-	0.948
	
	GATA (b)	0.944	0.972	0.916	-	0.944
	
	PITI	0.942	0.93	0.945	-	0.939
	
	GFI1	0.96	0.911	0.918	-	0.930
	
	HOXC	0.911	0.922	0.951	-	0.928
	
	OCTP	0.922	0.875	0.968	-	0.922
	
	EVI1	0.958	0.86	0.904	-	0.907
	
	TBPF	0.923	0.813	0.933	-	0.890

B	RBPF	0.944	0.943	0.961	-	0.949
	
	RORA	0.958	0.983	0.897	-	0.946
	
	HAML	0.943	0.935	0.943	-	0.940
	
	HOXF	0.884	0.889	0.884	-	0.886
	
	MEF2	0.905	0.885	0.775	-	0.855
	
	SRFF	0.697	0.717	0.681	-	0.698

C	MZF1	1.000	-	1.000	0.995	0.998
	
	ZFHX	0.984	-	0.984	0.984	0.984
	
	ETSF	0.983	0.982	0.983	-	0.983
	
	FKHD	0.962	-	0.962	0.962	0.962
	
	GATA	0.973	0.936	0.973	0.954	0.959
	
	IRFF	0.964	-	0.887	0.945	0.932
	
	CREB	0.938	-	0.938	0.914	0.930
	
	HOXF	0.975	0.870	0.975	0.857	0.919
	
	BRNF	0.946	-	0.906	0.899	0.917
	
	PARF	0.940	-	0.864	0.921	0.908
	
	ETSF	0.880	-	0.890	0.925	0.898
	
	OCT1	0.905	-	0.894	0.894	0.898
	
	SORY	0.879	-	0.879	0.927	0.895
	
	PLAG	0.900	-	0.882	0.887	0.890
	
	BRNF	0.810	-	0.810	0.916	0.845
	
	LHXF	0.839	0.846	0.839	0.849	0.843
	
	OCT1	0.846	-	0.841	0.820	0.836
	
	GZF1	0.761	0.858	0.858	-	0.826
	
	HNF1	0.801	-	0.803	0.819	0.808
	
	PAX6	0.778	-	0.769	0.781	0.776

D	SORY	-	0.991	0.987	0.986	0.988
	
	MOKF	0.983	0.983	0.983	-	0.983
	
	HOMF	0.989	0.950	0.989	-	0.976
	
	SATB	0.958	-	0.958	0.967	0.961
	
	CLOX	0.948	0.967	0.948	-	0.954
	
	CDXF	0.980	0.855	0.980	0.980	0.949
	
	PARF	0.921	-	0.921	0.995	0.946
	
	RBIT	0.924	0.965	0.924	-	0.938
	
	NKXH	0.933	0.928	0.933	-	0.931
	
	HOXF	0.923	-	0.923	0.942	0.929
	
	NKXH	0.946	-	0.835	1.000	0.927
	
	FKHD	0.922	-	0.922	0.909	0.918
	
	NFKB	0.864	0.992	0.841	0.947	0.911
	
	CREB	0.918	-	0.918	0.893	0.910
	
	MEF2	-	0.890	0.791	0.991	0.891
	
	OCT1	0.849	0.954	0.849	-	0.884
	
	OCT1	0.873	0.893	0.873	-	0.880
	
	SRFF	0.844	0.855	0.918	0.884	0.875
	
	HNF1	0.943	0.854	0.828	-	0.875
	
	PLZF	-	0.883	0.874	0.866	0.874
	
	PARF	0.860	-	0.865	0.897	0.874
	
	OCT1	0.862	-	0.856	0.899	0.872
	
	FKHD	0.867	0.861	0.836	0.919	0.871
	
	PARF	0.867	-	0.867	0.867	0.867
	
	BRNF	0.902	0.785	0.902	-	0.863
	
	CDXF	0.872	0.850	0.870	0.860	0.863
	
	MYT1	0.875	-	0.775	0.875	0.842
	
	BRNF	0.805	0.892	0.819	-	0.839
	
	CREB	0.844	-	0.833	0.833	0.837
	
	BRNF	0.796	-	0.807	0.889	0.831
	
	BRNF	0.790	-	0.790	0.898	0.826
	
	OCT1	0.790	-	0.783	-	0.787

List of matrix similarity scores (the similarity of each putative site to the canonical binding site for the relevant transcription factor) generated by MatInspector software for each putative transcription factor binding site in each species, for each region of homology. Matrix scores are ranked from the highest to lowest mean score.

The most frequently occurring transcription factor binding sites were those of BRNF and OCT1, which were represented in regions C and D a total of six times. PARF and FKHD binding sites were the next most frequent, represented four times between regions C and D. The HOXF family member binding sites were the most common, found in all of the regions and, in the case of region C, the site was conserved across four species. Eight transcription factor binding sites (HOXF, FKHD, SRFF, LHXF, CDXF repeated twice in the same region, MYT1, PLZF, and NFκB) were conserved across four species, and therefore displayed the highest level of conservation. With the exception of HOXF and LHXF (found in region C), all of these transcription factor binding sites at this four-way conservation level were found to localise to region D (Table 1 and Figure 4).

Region A showed nine areas of conserved transcription factor binding sites, the most common being GATA, occurring twice. All of the sites were conserved between bovine, goat and human. Transcription factor family members unique to Region A were EVI1, TBPF, HOXC, GFI1, PITI, and OCTP (Table 1, Figure 4).

Region B contained the fewest transcription factor binding sites of all the regions. Sites unique to this region were RORA, HAML and RBPF (Table 1, Figure 4).

Region C contained 17 transcription factor binding site family members, with three repeated twice (BRNF, OCT1 and ETSF). Although there appeared to be many conserved transcription factor binding sites, not all were present in the human sequence. Transcription factor binding sites that were conserved in humans are HOXF, ETSF, LHXF and GZF1, all unique to this region with the exception of HOXF (Table 1 and Figure 4).

Region D contained by far the largest number of transcription factor binding sites, with almost 50% of the total found. The majority showed conservation in the human sequence, and six sites were found to be very highly conserved across four species. CDXF sites are unique to region D and appeared twice close to each other conserved across four species. MYT1, PLZF, and NFκB were also unique to region D and showed conservation in four species. Other sites unique to region D and present in human were NKXH, MOKF, HOMF, RBIT and CLOX (Table 1 and Figure 4).

Many of the transcription factor binding sites identified in the sequences were found in clusters of two or more, adjacent to or overlapping one another. Region A transcription factor binding sites were localised to three clusters, with the largest harbouring five transcription factor binding sites. Region B had two clusters, Region C had five, two of which contained four sites each, and Region D contained nine clusters, although on average each cluster contained only two transcription factor binding sites (Figure 4).

## Discussion

The identification of gene regulatory regions through comparative genomics is a powerful entrée to directed studies of gene regulation. Using this method we have identified, for the first time, four regions upstream of *SRY *that show high conservation between human, bovine, pig and goat. Furthermore, these regions of homology share transcription factor binding sites that appear to be subject to strong evolutionary pressure for conservation and may therefore be important for correct regulation of *SRY*.

Mouse *Sry *5' sequences were found to be markedly dissimilar to other species across all regions of homology identified. This is perhaps not surprising given that mouse *Sry *coding sequences show particularly low homology to other species at the nucleotide and amino acid levels [7,20]. Moreover, mouse *Sry *is expressed for a short, specific time, with detectable levels of *Sry *transcripts first appearing at 10.5 dpc and waning by 13.25 dpc [21,2,23]. In other mammals, including humans, sheep, and pig, the gene remains actively transcribed into adulthood, albeit at a lower expression level than in fetal stages [24-27]. Therefore, mouse *Sry *evidently is regulated differently compared to other species and is therefore unlikely to have well conserved 5' regulatory regions.

Previous data bearing on the likely position of *SRY *regulatory elements has come from limited homology searches, transgenesis studies, and mutation analyses. Due to the unavailability of Y chromosome sequences from mammals other than mouse and human to date, minimal sequence has been available for homology studies. One study looked for conserved sequences upstream of *SRY *across ten species of mammal, including human, chimpanzee, gorilla, sheep, pig, bull, gazelle, mouse, rat, and guinea pig [28]. However, only 427 to 610 bp of 5' sequence was analysed, and no meaningful conservation was identified.

Boyer et al. (2006) used 3.3 kb and 5 kb of human *SRY *upstream sequence linked to human *SRY *coding sequence to produce transgenic mice, but only the larger fragment resulted in genital ridge expression of *SRY*. The same study showed that the pig 1.6 kb *SRY *promoter was sufficient for genital ridge expression [14]. Therefore we can postulate that the region necessary for genital ridge-specific regulation of *SRY *lies 5 kb upstream of the start of transcription in humans (corresponding to regions B, C and D from this study), and that this same site should be conserved in the pig 1.6 kb promoter (Region D). However, transgenic mouse models are subject to positional effects of the location of transgene insertion, which can cloud efforts to pinpoint gene regulatory sequences.

Two documented cases of mutations 5' of the coding region of *SRY *leading to pure gonadal dysgenesis have been reported in human. The first, a point mutation 75 bp 5' to the gene, was associated with male to female sex reversal. A nucleotide change from G to A, located in a motif conserved in primates, was found to be responsible [29], but this motif is not conserved in other species [30]. This mutation maps to region D of the present study. The second, a 25 kb deletion 1.7 kb upstream of human *SRY *was identified in a sex reversed patient [31]. The deletion would remove regions A-C and part of D, identified in the present study, supporting the hypothesis that regions A-D harbour important functional *SRY *regulatory elements, although the possibility that the deletion affects regulatory elements lying further 5' cannot be excluded as a cause of human sex reversal.

What transcription factor(s) may regulate expression of *SRY*? *SRY *is a master genetic switch that triggers testis development by initiating a cascade of gene expression. Its up-regulation marks the first male-specific gene expression event in the developing gonad. Therefore, any gene hypothesised to regulate *SRY *must be expressed equally in both sexes, before sex differentiation begins. *Sf1*, *Sp1 *and *Wt1 *are all expressed in genital ridges of both sexes and have been shown to influence expression of *Sry *in cell culture experiments [32-34]. Moreover, *Sf1*- and *Wt1*-knockout mice show gonadal sex development phenotypes [35,36]. Other genes known to have a role in gonadal formation and development, based on experiments in genital ridges and the absence of gonads in knockout mice are *Lim1 *[37], *Lhx9 *[38], and *Gata4 *[39].

The present study identified binding sites for a number of transcription factors 5' of *SRY*. The transcription factor families whose binding sites displayed the highest levels of conservation were LHXF, CDXF, HOXF, PLZF and NFκB. These families all have members that are plausible candidates for a role in *SRY *regulation. The highly conserved LHXF binding site found in region C could potentially bind either LIM1 or LHX9 transcription factors. *Lhx9 *is expressed in the genital ridges of male and female mice between 9.5 and 11.5 dpc. Gonads fail to form in mice null for each of these genes [37,38]. However, complete gonadal agenesis would implicate these genes in functions other than, or possibly additional to, regulation of *Sry*. PLZF and Nanog may bind to the HOXF and PLZF sites in the *SRY 5' *region, respectively. However, both are early germ cell transcription factors, and are therefore not present in the nuclei of supporting cell precursors in which *SRY *is expressed. NFκB is implicated in various stages of gonad development including spermatogenesis [40]. It is known to interact with AMH, and is likely have a role during the later stages of testis function, but expression in early gonadal development has not been described.

Perhaps most intriguingly, the two conserved CDXF binding sites in region D point to a role for CDX1 in *SRY *regulation (Figure 4). *Cdx1 *has been shown to be a direct target of retinoic acid [41], present in the gonads and mesonephroi of both sexes from an early stage [42,43]. *Cdx1 *is expressed in the mesonephros in the developing mouse embryo and remains detectable till 12 dpc. *Cdx1 *knockout mice are viable and show homeotic vertebral transformations [44]. In view of the present data, it will be useful to examine the gonadal phenotype of these knockout mice.

## Conclusion

In summary, we identified a large number of potential transcription factor binding sites localised to short regions of particularly high conservation in the *SRY *gene in human, bovine, porcine and caprine 5' flanking sequences. However, areas of high homology also exist that appear to lack binding sites for known transcription factors. These areas may also be important for the proper regulation of the gene by harbouring binding sites for unidentified proteins or transcription factors whose binding sites have not been characterized. The identification in the present study of regions of conservation upstream of *SRY *may facilitate the discovery of new mutations associated with human idiopathic XY sex reversal.

## Methods

### Bovine and goat *SRY *BAC sequence

The BAC clone RP42-95D10 from the CHORI BAC/PAC Resource Centre was previously identified as containing the bovine *SRY *coding region[17]. A 15 kb *Sry *fragment isolated from the BAC was cloned into pBluescript II KS+ using *Eco*RI, and shotgun sequenced by the Australian Genome Research Facility (AGRF) Brisbane, to five times coverage. The BAC clone (library number 568E7) containing goat *SRY *[18], was obtained from Dr. Eric Pailhoux. Primers designed from bovine sequences (MotAf, 5'-TCCTTCCTTTTCTCCTTTGTTG-3'; MotAr, 5'-TGGCCAAAAA CTACTTGATGA-3'; MotBf, 5'-GGAACAGGAGAGATCATGAAACA-3'; MotBr, 5'-CTTCACCATTCCCACTCACC-3'; MotCf, 5'-AACTTACATGCACTTCATTCCA-3'; and MotCr, 5'-GAGGACTTCA AATATTAATGTCATCAT-3') were used to amplify and sequence regions from the goat BAC. Assembly of goat sequences was performed using Sequencher version 4.6 (Gene Codes Corporation).

### Sequence alignment and binding site analysis

mVISTA  and SLAGAN (Shuffle-LAGAN) [45] were used for global alignment of the sequences after masking of repetitive elements. Conserved sequence blocks were analysed for conserved transcription factor binding sites using DiAlignTF software from Genomatix [46]. This analysis was carried out on the full-length conserved sequence blocks, as well as on core areas of high conservation found with ClustalW  within each block. Each block was first checked for sites conserved across four species, then three species. Only transcription factor binding sites that showed homology across more than two species were included in this report. Matrix similarity scores for the conserved binding sites were calculated by the MatInspector software from Genomatix [46].

## Abbreviations

BAC: bacterial artificial chromosome; bp: base pair; DNA: deoxyribonucleic acid; dpc: days post coitum; HMG: high mobility group; kb: kilobase pair; PCR: polymerase chain reaction; SRY: Sex determining region on the Y chromosome;

## Authors' contributions

DR, JB, PK and SL designed the study. DR executed all of the experiments. DR, JB, PK and SL wrote and proof-read the manuscript. All authors read and approved the final manuscript.

## Supplementary Material

Additional file 1 **Multiple sequence alignment of Region A, B, C and D with transcription factor binding sites**. ClustalW alignment of the four regions across human, bovine, caprine, porcine and mouse sequences with conserved transcription factor binding sites indicated using grey shading or boxing of relevant nucleotides. More detailed information on particular transcription factor families found per page are shown to the right of each alignment. Click here for file
