# Supplementary material for: New insights into SRY regulation through identification of 5' conserved sequences
Source: BMC Mol Biol. 2008 Oct 14;9:85. doi: 10.1186/1471-2199-9-85 (PMC2572636; doi:10.1186/1471-2199-9-85)
Supplement: Additional file 1 — Multiple sequence alignment of Region A, B, C and D with transcription factor binding sites. ClustalW alignment of the four regions across human, bovine, caprine, porcine and mouse sequences with conserved transcription factor binding sites indicated using grey shading or boxing of relevant nucleotides. More detailed information on particular transcription factor families found per page are shown to the right of each alignment. [file 1471-2199-9-85-S1.doc]

Region A

CLUSTAL 2.0.3 multiple sequence alignment

EVI1: Myleoid transforming protein

EVI1 ecotropic viral integration site 1 encoded factor, amino-terminal zinc finger domain. MEL1 (MDS1/EVI1-like gene 1) DNA-binding domain 1.

GATA: GATA binding factors

GATA, GATA1/2/3.

TBPF: TATA-binding protein factor

ATATA avian C-type LTR TAT box, LTATA Lentivirus LTR TAT box, MTATA muscle TATA box, TATA cellular and viral TATA box elements, and Mammalian C-type LTR TATA box.

HOXC: HOX – PBX complexes

HOX/PBX binding sites, PBX1, PBX-HOXA9 binding site.

HOXF: Factors with moderate activity to

homeodomain consensus sequence

Barx2, CRX, GSC, Gsh-1/2, HOX1, HOXA9, HOXB9, HOXC13, NANOG, OTX2, PCE1, PHOX2a/2b, PTX1 pituitary homeobox.

GFI1: Growth factor independence transcriptional

Repressor

GFI1.01/02, GFI1B.01.

PITI: GHF-1 pituitary specific pou domain TF

Pit1, GHF1.

OCTP: OCT1 binding factor (POU-specific domain)

OCT1P Octamer-binding factor 1, POU-specific domain)

bovine_A -----------------------------------------------TTTGGAACTCTGC 13

goat_A ------------------------------------------------------------

human_A AATTTCAAATAACATATAATTGCTAGGTAATACAAAAATTTGAAAAGCCCGGAATTCCTC 60

mouse_A ------------------------------------------------------------

bovine_A ATTAACATGTATATATCCTTCCTTTTCTCCTTTGTTGTAGTTACAGCAAAAAAATTAATT 73

goat_A ----------------------------------------------------AATTAGTT 8

human_A AG-ATTTTTCCTGTTGTGGTTATTGGATACTTGCTGCAGGTTTCTGCATAAAGGCTATTT 119

mouse_A ----------------------------------------------TCTTTTAACTGATT 14

* **

bovine_A CACAGAGAAGCAATATTTTTCAGCTTTTAAGACACTGTAACCATTGTTTTTGAAACAAGA 133

goat_A CCCAGNGAAGCAATATTTTTCAGCTTTTAAGACACTGTAACCATTTTTTTTGAAACCAGA 68

human_A TACAAACAAGCAACATTTTTTAGCAGT-AAGATACTAAAGCCATCGTCATTCAAAGAAGG 178

mouse_A TACAGA-AAACAATACATGTAAGTTAC-TGTTCACAATA--CATAATTTTGAAAATCA-A 69

** ** *** * * * ** ** * *** * * *** *

EVI1 GATA

bovine_A CCAAAACCGGGATTCATGTTATATCCATTATTACTAAAGTACTGTTCTGAAGATTTAAAG 193

goat_A CCAAAACCGGGATTCATGTTATATCCATTATTACCAAAGTACTGTTCTGAAGTTTTAAAG 128

human_A CAAAAACGGGCA--CAAATTATAATAATCATTGTTATATTATTGTTTTGAAGGTT--AAG 234

mouse_A CTAAAACTCAAA--GGTCTTAAGACCATG-----TAGAACAGTAGTTTAATTGTTTCCAA 122

* ***** * *** ** * * * * * * * ** *

bovine_A ATGAAAAGGATAAGTATAAATATACAGTGCTTATGAAAAAAGTGTCAGTTA-AATGTATG 252

goat_A ATGTTAAGGATAAATATAAATATACAGNGCTTATGAAAAAAAAANTA-TTA-NATANNTG 186

human_A ATAAAAAGGATAAATGCAATTTTAAGTCCAAAATCAGGAAAGTATTTTTGA-AATGTATG 293

mouse_A GAGTTCAATACATCAGATAATACACCATGTTGTTGTATAGTATTTAGTGTATAAGATGTC 182

* * * * * * * * * * *

GATA TBPF HOXC HOXF GFI1

bovine_A TCAGCTAAA-GGATGATATCTTTCATATATAAATCACTACAGGAGAAAGAAAAATCCTCA 311

goat_A TTTATCANATAAANAATATCTTTCATGTATAAATCATTACAGGAGAAAGAAAANTCCTCA 246

human_A ACAGCTAAA-GGATAATATCTCTTAACTATAAATCACTAAAGGA---ACAATATTTTTCA 349

mouse_A ACAGTTGAG--------------------------------------------------- 191

* ****** * * ********* ** **** * ** * * ***

PITI1 OCTP

bovine_A TCAGAAAACTGAGAAATATCCATAAGAACTGACCAATTATTAGGGTACTAAA---GGTAT 368

goat_A TCAGAAAACTGAGAAATATTCATAAGAACTGACTAATTATTAGGGTGCTAAA---AGTAT 303

human_A GAAGAAAAAT-AGAACTATTCAT--GATTTGACCAAGCCTTTGGACACTAAACAAAATAT 406

mouse_A ------------------------------------------------------------

****** * **** *** *** ** **** ** ** ** ***** ***

bovine_A TTCAAAAATAAGGAGCTGAATGAAAACAGTTTCTGATGTCAATAAAATGAG----GAAAA 424

goat_A TTTAAAAATAAGGAACTGAATGGAAAGAGTTTCTGATGTCAATAAAGTGAG----AACNA 359

human_A TTCAAAA--CATTAATAAAAAGAAAGCAGTCTCTAATCTTAATGCAACAGACTGAGAAAA 464

mouse_A ------------------------------------------------------------

** **** * * ** * ** *** *** ** * *** * * *

bovine_A AATAGTGTATACAATG-TACAGTAGAGCAAGTCCATGGAATATCATTAATAAATTAACAA 483

goat_A AATAATATATACAAGG-TANAGTA------------------------------------ 382

human_A AATAATGTACGGAAGGGTACAACAGAATGCTTCCATGAGGTATCTTTAAAAACTG----- 519

mouse_A ------------------------------------------------------------

**** * ** ** * ** * *

Region B

CLUSTAL 2.0.3 multiple sequence alignment

bovine_B CTGTCTCCTTGAAAGACTTACTTTCAAACAGGGGCAAGAGCCGAGACCACTTTACTTTTA 60

goat_B ---------------------TTNTNTCCAGNGGCAAGAACCAAGNCCNTNATNN----A 35

porcine_B ------------------------------------------------------------

human_B ------------------------------------------------------------

mouse_B ------------------------------------------------------------

bovine_B GCCTGTAGCCCACTGATGGTCTAGTGGCTAGGACTCTTTGTTTTCATGCTGGCTGATGAA 120

goat_B GCCTNTAGCC-GCNGAAATTNTNNNGNNTAGGACAATANGNTTTCATTNTANTTNATGCA 94

porcine_B ------------------------------------------------------------

human_B ------------------------------------------------------------

mouse_B ------------------------------------------------------------

bovine_B AGTTTAATTTCTGACCAGTGAATTAAGATCTTTCTTCAGGAGTACTCACTGCTCTCCCTT 180

goat_B ANACTAATTCNNGNNCAGTGAATTGNGATCTTTCNTCAGGNCTNNTCCCNGTTCTCCNTA 154

porcine_B ------------------------------------------------------------

human_B ------------------------------------------------------------

mouse_B ------------------------------------------------------------

bovine_B CAAAATCAGATTCACTTCAGAACTTAGACTATTAT---TGACTTTCATAGGAACAGGAGA 237

goat_B CAAGATCANNTTCATTTCAAAANTGANACNANTATAATTGACNTTCATAGAAACAGGGGA 214

porcine_B ------------------------------------------------------------

human_B ------------------------------------------------------------

mouse_B ------------------------------------------------------------

RORA: v-ERB and RAR-related orphan receptor alpha

REV-ERBA orphan nuclear receptor rev-erb alpha (NR1D1), RORA/RORA1/2 RAR-related orphan receptor alpha/1/2, RORGAMMA RAR-related orphan receptor gamma, VERBA viral homolog of thyroid hormone receptor alpha1

MEF2: Myocyte-specific enhancer binding Factor

MEF2, RSRFC4 related to serum response factor, SL1 member of RSRF

HOXF: Factors with moderate activity to

homeodomain consensus sequence

Barx2, CRX, GSC, Gsh-1/2, HOX1, HOXA9, HOXB9, HOXC13, NANOG, OTX2, PCE1, PHOX2a/2b, PTX1 pituitary homeobox.

RORA

bovine_B GATCATGAAACAGATAAGAGATCAAAAGTGACCTTCTTTTTTCAAGAAAAGAACAAGTTA 297

goat_B GATTGTGAAACAGANAAGAGANCAAANGTGACCTGTCTTTTT-----AAAGAACAAGCTA 269

porcine_B ------------------------------------------------------------

human_B ---------------AAAAGATCAAAAGTGACCTTCATTTTATG-GAGAGAAACAAGCTA 44

mouse_B ------------------------------------------------------------

** *** **** ******* **** *********

bovine_B AGTCAAACAACATCTGAGCTGTCTAGAGGAATTAAAAATGGTAGCTTGTGCTTTGTGCAT 357

goat_B AATCAATCAACATTTGAGCTGACTAGACAAATTAAAAATGGTAGCTTGCGCTTTGCGCAT 329

porcine_B ------------------------------------------------------------

human_B TAACATGTAGTATCTAAGCTGATTAGAAGAACTAAAAAGAGAAGCTCATACTT-GTGCAT 103

mouse_B --------------------------------------ATGTCTCTTTAAACTTGTTTGT 22

** * ** * ***** **** ** ****** ** *** *

bovine_B CGGAAATTAAATGGCAGGAGTGGGACCATTTTTAATGTTTCAAGGTAGAAAGGAAGACTG 417

goat_B TGGAAATTAAATGGCAGGAATCAGAGCATTTTTAATGTATCAAGGTAGAAAGGAAGACTG 389

porcine_B ------------------------------------------------------------

human_B CAGAAGGTAAATGA-AAGAGTGAAGTTACCTCTT-TGTTTTAAGGAAGAAAGGAAAATTG 161

mouse_B AAGAACAAGAGTGGGTAACTTTGAACCATTCCTTAGCTTAACAATCTGGCAGTTGAGTTA 82

*** * ** * * * * * * ** *

bovine_B CTTTGAAATGGGCTTTTTTCTCTTTATTTTAATATCAACACTTACAAATAAGCTAGGAAA 477

goat_B GTTTGAAACTGGCTTTTTCCTCTTTATTTTAATACCAATAGTTACAAACAAGCTAGGAAA 449

porcine_B -------------------------------------------------------CATTA 5

human_B --TGGATGTCATCTGTTTTCTGTTTACAT-----------ATTTCAGGCATGGATAGCCA 208

mouse_B ATGTGCAGATGGCCATTCATTCATCCCACATATACTTGCCCTCCCCTTCACCTTTTGTTT 142

* * * ** * * * *

bovine_B ATACATCAAGCTATAGTAATTTTAAAGTAATTTCCTATATTAGAAATGTCAAAA-TGGTA 536

goat_B ATACATCAAGCTATAGTAATTTTAAAGAAATTTCCTATATTGGAAATGTCAAAA-TGGTA 508

porcine_B AAAGATGGTCCTGGGAAAACTGTAAAGGAAATGGACATGTGAGTGCACTTAGGT-TAGTA 64

human_B CAATGTGATTTTAAGACGGTT--AGTTACAACTGATTTGAAAAAAAAAAAAAAT-GCTTC 265

mouse_B TATTCCAATCATTTTGTATAT---GTGGTGTCTTAAGTGTTGGTACATCCACTTAGCTTG 199

* * * * *

MEF2

bovine_B AAATTAAAAGAAAACATTAAAATGATGCTAGAGAAGAAAGCAAGAAGCATT-ATTTT-TA 594

goat_B AAATTAAAAGGAAACATTATAATGATGATAGAGAAAAAANCAAGAAGAATTTATTTT-TT 567

porcine_B AACT-----ACAAGTGGTGTAA-GATTTGAGGGGGTTATTTTACAATCACATATATT-CA 117

human_B ACTCTATGAGAAATTTCTTCCCAAGTATGAAACCTTGTTTTTACAGGCAAT--TTCC-TA 322

mouse_B ACTATTTGTCCATTTTGTTTCTGATTCTTAGCTAGCCCTTGAAAACACAGCTTTGCTGTA 259

* * * * * * * * *

HOXF

bovine_B CAGTTGTAAACCAGATATG-TATTTATATAA-ATAGCATATATGTATTTATATAAATAGC 652

goat_B CAGTTGTAAACCAGATAGG-TATTTATATTTTACAGCATATATTTATTTGTA-----AGT 621

porcine_B TA-TTTTGATGGGGATGTCCTGATAAAATTAAAGGAAATTAATGTGGTAATAGTCATGTC 176

human_B TACTT-TGAAAAAATCAAAATAATAAAGTAAAAGAAAAATAATTCAGGTGAAGTTAGAGA 381

mouse_B TGTCAATAAAACAGTCTATTCTTTAGAGATAGATAATAAAAGAGAAGCAAAATGTGACTC 319

* * * * * *

bovine_B ATATATGTATTTGCAAGTTTCTTTTTTTCAGGTGGTTTCTATTTCTTCCACCCCATA-TG 711

goat_B TTCTTTCTTTCT-----TTTTTTTTTTTCNGGGAATTTCNGTTTCNTCCCCCCCNAAANN 676

porcine_B ATTTTCATTGTATGCATTTCTTAATTAAAAAATGTTCTGAATTTTTTGTGTGTGTATGTG 236

human_B AAAAAACAGGCA-----GCATTATTTTAAAGTTGTAAACTATTTTGTTTACTTATAGTTT 436

mouse_B CTTATGCATAAAGGCTTATTTTA-CCAAAAGATATAGAGAGCTCATAGCATAGTTCATAG 378

* *

bovine_B AATTAAGATGCTCCT-GGA-AGGTGATAACAATT-AAATCAATCATGTTTATGGG-ATTC 767

goat_B AATTANANNGGNCCNAGGA-AGGGGAAAANAATGGAAATCCATCCTGNTTATGGGGATCC 735

porcine_B TGTGGAGGGGGTGAGTAGA-GGAGGAAAAAAATTAAAAAAAATTATTAATATAAA-ATT- 293

human_B AATTTACATGT---------AGTAGATATGCATTTGTAAGGTTCTTCGGCTCAGGTAGGA 487

mouse_B AACAACTGGGCTTTGCACATTGTGGAGGAGAACT--AAAATGTCATACACACACACACAC 436

* * ** * * * * *

bovine_B AGATTCTATATGACTTATTTAAGTCTTGGAACAATGAGATAA-TTGAGTTTTGATATGTA 826

goat_B NGATTCTATATGCCTTATTTAANTCTTGGAACAAAGNGANAAANTCCGTTTTTNTNCATA 795

porcine_B ------------------------------------------------------------

human_B GATCATTCTATTTCCCACTGCACCCT----ACTTCATCCTCCCACTGGCAAATAATTAGA 543

mouse_B ACACACACACACACACACACACACAC----ACACACACACACACACACACACTCACAAGA 492

* ** *

bovine_B TCTAACACATTAAAAAGATATTTCATTTTAATTCATTAAGTCCAGGGCATGTTTAAAAAT 886

goat_B TTTNAACNTCCAAAAAGGGATATTNNTTTNATTNCTTAANTNCCGGCCATGTTTNAAAAT 855

porcine_B ------------------------------------------------------------

human_B TTATCCCTGGGAAAAAAAGATGCCAGTAAAATTGATCATGTTTAA---ATGCATCAGTTG 600

mouse_B CTTTACATACTCATGATGTAGTTTTGTGTGCTAGAGAGAAACCCTGATAAACAGAAAGAT 552

* * * * * * *

bovine_B CCAGCAAGATTAAATAAA-------GAAACAAATGTTCTGTTTATCATTAAAAGTGAAAG 939

goat_B CCCGCNAGNTTAAATNAATTTAAAAAAAAAAAAAGTTCTGTTTATCATTAAAAGTGAAAG 915

porcine_B ------------------------------------------------------------

human_B CTAGGTGATTTATCTGATT--AAGTCTTGAAACAGTAGAACCTAGCAATTAAAGTGAGCA 658

mouse_B GCA--------------------------------------------------------- 555

* *** * ** ** ** ** * *******

bovine_B TGAAGGTTGCTCAGTCATGTCTGAC--TCTTTATGACCCCATGGACTATACAGTACCATG 997

goat_B TGAAAGATGCTCAGTCATGTCTGAC--TCTTTGTGACCCCATGGNNTATATAGTCCCATG 973

porcine_B ------------------------------------------------------------

human_B TTAACTTCTACCTACCAAATCAGAAGACTATTCTAACTTTTTGAGAATTAGATGTTGAAA 718

mouse_B ------------------------------------------------------------

* ** * ** ** ** ** ** * * *

bovine_B CAGTTCTCCAGGCTAGAATACTGGTGTGAGTAGCCATTCCCTTCTCCAGGGGATCTTCCC 1057

goat_B CAGTTCTCCAGGNTAGAATACTGGAGTGGGTAGCCATTCCCTTCTCTAGGGCATATTCCC 1033

porcine_B ------------------------------------------------------------

human_B ATATGGCCCATGAATTTAGCATGGTTAAAATAAAAAA---CATGCAAACAAAACAAACCC 775

mouse_B ------------------------------------------------------------

* *** * * *** ** * * * * * *

bovine_B AAGGCAGGTATCAAACCCAAGTCTCCTACATTAAAGGCGGATTCTTTACCAGTTGAGCCA 1117

goat_B AACACAGGGATCAAACCCAAGTCTTCAGCATTAAAGGGGGATTCTTTACCAGTTGAGCCA 1093

porcine_B ------------------------------------------------------------

human_B AACATCTTGAAAGGACATTTGACTCTAAAGTCCCAAAAATAATCACAAGTCTAAAAATCC 835

mouse_B ------------------------------------------------------------

** * ** * ** * * * ** * *

bovine_B CAAGGGAAGTGCAAGAATACTGGAGTGGCTGGCCTATCCCTTTTCTAGTGTATCTTCCCA 1177

goat_B CAAGGGAAGTGCAAGAATACTGGAGTCTGCGGCCTATTCCTTTTCTAGTGTATCTTCCCA 1153

porcine_B ------------------------------------------------------------

human_B TAAGTTTAGTGTTACTCTATTACA--------CCTTTTTATTTGTAAGTGTCCTTTCACA 887

mouse_B ------------------------------------------------------------

*** **** * ** * * *** * *** ***** *** **

bovine_B AACAATAAAATGAACTGGGACCTCCTGATTACAGGTGAATTATTTACCAAATGAGCTAGC 1237

goat_B AACAAGGAAATTAACTGGGACCTCCTGATTACAGGTGAATTCTTTAACAACTAAGCTAGC 1213

porcine_B ------------------------------------------------------------

human_B A---AAGTTTTAAATTTTGCTCTTGTGCATTTTATTTACCTTTTCTTTTGTTGTTTGTGT 944

mouse_B ------------------------------------------------------------

* * * ** * * ** ** * * * * ** * *

bovine_B TATCAGGGAAGCTCTATGTATTATTAGAGTTAAAAAAAAAAAAAAAAACGCAGTTCACAC 1297

goat_B TATCAGGGA-GCTCTATGTATCATTAGAGTTAAACAAACAAACAAAAACAGCTCACAGAC 1272

porcine_B ------------------------------------------------------------

human_B CTTTGGTGA---CCTGCCAACCATTAGACTTCAA----------AAAACAGCCTATAGCC 991

mouse_B ------------------------------------------------------------

* * ** ** * ****** ** * ***** ** *

bovine_B A--CTAGGAAATAGGTGGACACATAAATTGATATAGATTAGTAAACTACAAATAGGGCAA 1355

goat_B AAACTAGGAAATAAATAGACACATAAATTGATATAGATTAGCAAAATACAAATAGGGCAA 1332

porcine_B ------------------------------------------------------------

human_B AAGCTGCAGGATAAATGAACACATAAGTTGACTTAGAATAGTCAACTCTGTCTAGTATAC 1051

mouse_B ------------------------------------------------------------

HAML: Human acute myelogenous leukemia factors

AML1/CBFA2 Runt domain binding site, AML3 runt-related transcription factor 2/CBFA1

SRFF: Serum response element binding factor

SRF.01/02/03

RBPF: RBPJ kappa

Mammalian transcriptional repressor RBP-Jkappa/CBF1

* ** *** * ******** **** **** *** ** * ** *

HAML SRFF RBPF

bovine_B GATTTAGTGTCCTTATATTTTAACCACATAATTTCATATGTTTTGCTGGGAAATGTCCTG 1415

goat_B GATTCAGTGTCCTTATATTTTAACCACATACTTTCATA--TTTTGTTGGGAAATTTCCTG 1390

porcine_B ------------------------------------------------------------

human_B AATTTATGGGGGATGGTTTATGACCACATATATTTCTAC--TTTGATGGGAATATCTTGA 1110

mouse_B ------------------------------------------------------------

*** * * * ** * ******** ** ** **** ****** *

bovine_B ATAGTATTAGA-GGACATAAATGGAGCAAAATCTGTGCAATTATAATTGTATCTATTCCT 1474

goat_B ATAATGTTAGAAGGACATTAATGCAGCAAAA----------------------------- 1421

porcine_B ------------------------------------------------------------

human_B ATAAAATTAGA-GAGAATGAGTGGAGTAATATTCACAACATTTTTGCTGCATTCATCCCT 1169

mouse_B ------------------------------------------------------------

*** ***** * ** * ** ** ** *

bovine_B TCATTTGAAAACGTTCTAACTATTTTGTGGGGGGAGGTGAGTGGGAATGGTGAAGAGAAA 1534

goat_B ------------------------------------------------------------

porcine_B ------------------------------------------------------------

human_B GAATTTGAAGAAATACCAAAGTACATCTTGTGAGGAGAAAAAATAAATAAATTCATATAA 1229

mouse_B ------------------------------------------------------------

Region C

CLUSTAL 2.0.3 multiple sequence alignment

bovine_C GAGTGACTAAAATGAACTGATAACTTACATGCAGTTGATTCCATTGCAATAATCTAGAA- 59

goat_C -----------------------------------------CATTGTAATAGGCTAGAAA 19

porcine_C --------------------TAGCACAAATACACTTTATTTCATTGGAATAATCTAAAT- 39

human_C -------------------GTACCTCAAATGCAATTAATTGCATTGGACCAATCTAAGT- 40

mouse_C ---------------------------------------------------AAATGAAC- 8

*

bovine_C TATAGT-GTTCAGTTCTTATTCATTTTT-ACT-------------TTAATTTGAGAAGAA 104

goat_C TATAGNAGTTCAGTTCTTATTCATTNTT-ACT-------------TNAATTTGAGAAGAA 65

porcine_C TATCAT-TTTCAGTTCTCGTTTATTTTTTATT-------------CTAAGTTGAAAAGGA 85

human_C TACTATTCTTCAGTTTTCATTTTTATTTCATTATTCATTTCATTTTTATTCTGATATAAA 100

mouse_C TACTGCATCCCAGTCATTAGA-ATGCTCAACCTGGA---------TGATTTTTACCATGA 58

BRNF: Brn POU domain factors

BRN2/3/4/5

HOXF: Factors with moderate activity to

homeodomain consensus sequence

Barx2, CRX, GSC, Gsh-1/2, HOX1, HOXA9, HOXB9, HOXC13, NANOG, OTX2, PCE1, PHOX2a/2b, PTX1 pituitary homeobox.

LHXF: Lim homeodomain factors

LHX3 and LMXB1

GATA: GATA binding factors

GATA, GATA1/2/3.

ETSF: Human and murine ETS1 factors

c-Ets-1/2(p54), ELF-2(NERF1a), ELK1, FLI, GABP GA binding protein, GABPB1 GA repeat binding protein beta 1, NRF2 nuclear respiratory factor 2, PDEF Prostate-derived Ets factor, PEA3 polyomavirus enhancer A binding protein 3, ETV4, PU1, SPI1, SpiB.

IRFF: Interferon regulatory factors

IRF1/2/3/4(NF-EM5, PIP, LSIRF, ICSAT)/7, ISRE interferone stimulated response element.

OCT1: Octamer binding protein

OCT1/2/3

PAX6: PAX-4/PAX-6 paired domain binding sites

PAX4 and PAX6 paired domain binding site

MZF1: Myeloid zinc finger 1 factors

MZF1

GZF1: GDNF-inducible zinc finger gene 1

GZF1(ZNF336)

HNF1: Hepatic nuclear factor 1

HNF1

FKHD: Fork head domain factors

FHXA/B, FKHRL1 (FOXO), FREAC2/3/4/7 fork head related activators (FOXF2, FOXC1, FOXD1, FOXL1), HFH1/2/3/8 (FOXQ1, FOXD3, FOXI1, Freac-6. FXF1), HNF3B (FOXA2), IlF1 (FOXK2), XFD1/2/3.

ZFHX: Two-handed zinc finger homeodomain

transcription factors

AREB6 (Atp1a1 regulatory element binding factor 6), deltaEF1 (Delta-crystallin enhancer binding factor, transcription factor 8, zinc finger homeobox 1a), SIP1 (Smad-interacting protein)

PARF: PAR/bZIP family

DBP Albumin D-box binding protein, HLF hepatic leukemia factor, TEF Thyrotrophic embryonic factor, VBP PAR-type chicken vitellogenin promoter binding protein.

CREB: Camp-responsive element binding

Proteins

ATF, ATF2/6 activation transcription factors, c-Jun/ATF2 heterodimers, CREB, CREB1/2, CREB2/cJun, E4BP4, TAX/CREB complex, XBP1 X-box-binding protein.

** **** * * * * * * * *

BRNF HOXF/LHXF GATA

bovine_C ATAAGAATCTATAATATGAAAAATTATTTTAATCTAATATCTCTGGTGCATTTTCCTTTT 164

goat_C ATAAGAA-CTATAATATGAAAAATTATTTTAATCTAATATCTCTGCTGCATTTTCCTTTT 124

porcine_C ACCAGAC-----CATACATGAAATTACTGGAATCTGATATCTCTTGAGCATTTTTCTACT 140

human_C AATGAACCAGGATCTGTGTGAAATTATTTGAATCTAATGTCTTT-GAACATTTTTCT--T 157

mouse_C AAGAATT---------------TTTGCTATTATGTAAGAAATTT-GTATACATTACTTTT 102

* ** ** * ** * * * * * ** ** *

bovine_C CTAGAATATTAATCCCACC--AAAACAGAAATTGAGTTTGATAGTTTGGCAAACTTTACT 222

goat_C CTAGAATGTTAATCCCACC--GAAACAGAAAATGAATTTGATAGTTTGGCAAACATTGTT 182

porcine_C GCAAAGCTGTAATCTTCCCCAAAGAAAAAAAATGAGTTTGTTAGTTGGACAGCATTTATT 200

human_C ACCATACCTTAAGATTAAA--AAAACAAAAAA-AAATCCCTTAGTTTGGCAACTTTTGCT 214

mouse_C ATGATGCATTGATATGACCAAGAAAGGAAGCAAAAGTTCTTTTATGGTGTAGATATTTAT 162

* * * * * * * * * * ** *

ETSF

bovine_C GTTAGTGAATCCCCTTTGGGTTTAACTTTTTAAGAACTGTATCACTTCCTACTA-CT--- 278

goat_C GTTAGTGAATCCTCTTTGGGTTTAACTTTTTAAGAACTATATCACTTCCTACTA-CT--- 238

porcine_C CTCAGTAAATTCCATTTGGATTTAACATTGGTAGGACTTACTCCCACTTTGCTT-TTGGG 259

human_C GTTGGTTAAGCCCGTTTGGATTTAACATTGACAGGACCAGCTAACTTCCTACCAGTTAAC 274

mouse_C ACTGGGCTATAGAGCCAGAACAGACTATTTC-----TTAGTTTTTTCCATGCTGCCTG-- 215

* * * * ** * * * *

bovine_C -TTGCTTGTGGG------------GATGAT--TGAG-----------ACATAGTAA---- 308

goat_C -TTGCTTGTGGG------------GATGAT--TGAGGAAAAATTGAGACATAGTCAGTCA 283

porcine_C TCTGATTGAGGA------------AAAGATATTTAACAACTGTTGGGACATAGCCAAAAA 307

human_C ATTGCTTGTGGGCCTGACTGATGTAAAAATATTTAACAACTGTTGAGAAATAGTCATCAG 334

mouse_C --CCGTAGTAGAC-----------TATGATACTAATGATAGGTTCCATGCTTATCATTTT 262

* * * * ** * * * *

IRFF/OCT1 PAX6

bovine_C TGAAAAGGAAATTT-ATAGTCATTTAACCCAATA--ATGCCGAGAA-----------TAA 354

goat_C TGAATAAGAAACATGATAGTTATT-AACCCAACA--GAGCCAAGAA-----------TGA 329

porcine_C TGAAAAAGAAAACTGGTAGTCATTTAACACAATA--GTGTCTTAAAATTTTCTTTACTGA 365

human_C TGAATCTTAAAGGTCGTACTCATATAAAACAATATAGCGCTTTAACTTTTTATTTACTGA 394

mouse_C TAACCCTT---TCCACTACTTTTGCAAGCTC----------------------------- 290

* * ** * * ** * * * *

MZF1

bovine_C ATGTGGGGAATGGGTGTGGTATCCTAG---------TCACCCAAAAGCTCAGGATAGCAT 405

goat_C ATGTGGGGAATGGGTGTGGTATCCTAG---------TCACCCAAAAGTTCCGGGTAACAT 380

porcine_C ACATAGGGGATGGAAATGGCATTCCAG---------TTACCCAGGAGTTCAGGCAAGCAT 416

human_C ATTTGAGAGAAAGGAATAGTGTTCCAGAAACTGGGATCACTCAGGCGCTCAGGGAAGCAT 454

mouse_C ------------------------------------------------------------

* * * * * * * * * ** * ** ** * ** ** * ***

GZF1

bovine_C ACATTTTGTCGCTACATTTTGTATGAAGGTGCAATATTATAAGAATTACTAAATATTGTA 465

goat_C GCACTTCATCTCTA------GTATAAAGGTGCAATATTATAAGAATTGCTAAATATTGTA 434

porcine_C ATTTGCACTCTCCA------GAATAAAGACTCAATGTTCTAAGAACGGTTAAATATTGCA 470

human_C ATGGTTAGCTGATG------ATATAAAGGCCCAATATTGGAAGAACTGTTAATGTAGTCC 508

mouse_C ------------------------------------------------------------

****** *** ** ***** ***

HNF1

bovine_C TGTGAGCATAGTGACTGAGT-ATATATGAGGCTTCTGAAATAAAAATAAGCTGA---AAC 521

goat_C TGTGAATATAGTGACTAAGT-ATAT--CAGGCTTCTGAGATAAAAATAAGCTGA---AAT 488

porcine_C CCTGGGCACAGCAGCTGAGT-ATCAGCCATGCCTGTGAAATAAAAATGAGTTGA---AAC 526

human_C CCTGGAGACAGTGAGTGAATTATTAGCCCTGGTTGAGAGATTAAAATGTGTTGATATGAT 568

mouse_C ------------------------------------------------------------

** * ** * * ** * * ** ** ***** * *** *

bovine_C ATTAAAAATG-------------------------------------------------- 531

goat_C ATTAAAAATG-------------------------------------------------- 498

porcine_C ATTAAATATGTACTTATAAATTCCCTGGGTAAGCTCAGTGGGTTAAGGAGCTGGCATTGT 586

human_C ATTATAGAGGCAAATTTAATGCTCTCGGCCTCCCCCTGCA--------TCCTTATTTTTG 620

mouse_C ------------------------------------------------------------

**** * * *

bovine_C ------------------------------------------------------------

goat_C ------------------------------------------------------------

porcine_C CACTGCTGTGGCTTTGGTTACTGCTATGGTGGGGGTTCAATTCCTGGCCAGGGAACTTAA 646

human_C CATGTAGGTTTATTACTTTACAGGAGAACCAACACTTTTTTCCTTTTTTTTTTTTTAAGT 680

mouse_C ------------------------------------------------------------

FKHD/ZFHX PARF/CREB

bovine_C -------------------CACTCAAAGAAACATT-AGGTAACCTACTGATAT----ATT 567

goat_C -------------------CACTCAAAGAAACATT-AGGTAACCTGCTGATAT----ATT 534

porcine_C AAAAAAAATAAATAAAAGGCACTCAAAGAAACATT-AGGTAAACTGTTGATATCA-TATT 704

human_C GGTTGAGTTTGTTTTTCTGAACCCAGAGGCATGTTTACACATTTCATTAATTTAAGTACC 740

mouse_C ------------------------------------------------------------

** ** ** * ** * * * ** * *

PLAG ETSF

bovine_C ATTGAGATGGACT-ATAC--ACCCTTTCTTCATCCTCTTCCCTCCATTCT-TGCATCAAA 623

PLAG: Pleomorphic adenoma gene

Pleomorphic adenoma gene (PLAG) 1, a developmentally regulated C2H2 zinc finger protein

ETSF: Human and murine ETS1 factors

c-Ets-1/2(p54), ELF-2(NERF1a), ELK1, FLI, GABP GA binding protein, GABPB1 GA repeat binding protein beta 1, NRF2 nuclear respiratory factor 2, PDEF Prostate-derived Ets factor, PEA3 polyomavirus enhancer A binding protein 3, ETV4, PU1, SPI1, SpiB.

BRNF: Brn POU domain factors

BRN2/3/4/5

OCT1: Octamer binding protein

OCT1/2/3

SORY: SOX/SRY-sex/testis determining and

related HMG box factors

HBP1, HMGA1/2, HMGIY, SOX5/9, SRY.

goat_C ATTTAGATGGACT-ATACTGGCCCTTTCTTCATCCTCTTTCCTCCATTCT-TGCATGAAA 592

porcine_C AACAAGGTGGACC-ATGCC-TCCCTCTCCCCCTCCCCTTTCCTCCATTTT-TGAATAAAA 761

human_C AGAGAAGTATACTGAGAACCTACTACATGTCACACACTATCCTAGGAGCTGTAGACAGAA 800

mouse_C ------------------------------------------------------------

* * * ** * * * ** **** * * * **

bovine_C AGTCTCTAC--TTTAGAGAATC-ATGGAAGCTTT-GGCTTTTTATAAGAGGAATACCAGT 679

goat_C AATCTTTAC--TTTAGAGAATC-ATGGAAGCTTTTGGCTTTTTATAAGAGGAATACCAGT 649

porcine_C AGGTGTTAC--TTTTGAGACTC-ATTGGGGGTTTTGTTTGTTTGTTTTAAGTGGATTAAC 818

human_C AAATAATAAAATCTCGTGTCTTTATTTAAATTTT--GTTGACTGCCACCCCCAAGTGAGT 858

mouse_C ------------------------------------------------------------

* ** * * * * * ** ** * * *

bovine_C TTGT---TTGTTTGTTTTTTTTTTCT---ATATGCTTTTAGCCATTTT-GACTCTACTTG 732

goat_C TTTTGTTTTGTTTTATTTTGTTTTGTTTCATAAGCTTGGAGCTGTTTTTGACTCTGACTC 709

porcine_C C--------ACTTTCTTTCTTTCTCC-----AGGCTTGGAGCTATTTT-CACTCCTTTTG 864

human_C CTCC-TCACACACCCCGTCCTGTAATATATGCATCTGGGAGGTCTTTTTTGCCTTCTTAA 917

mouse_C ------------------------------------------------------------

* * ** ** ****

bovine_C TTGATTGAAGTATATTGTACAGGTAT-TTCAAGAAAGTAATATATATGCCAGGTTGTATT 791

goat_C TTGATTGAAGTATATTGTAGAGATAT-TTCAAGGAAGTAATATATATGCCAGCCTGTATT 768

porcine_C TGATTTGAAGCA--TTGAAGAAATAT-TGCAAGGACCTAATGTA--TGCCAGGCTCTATT 919

human_C AAACATATAGATGGTTGGACATATGTATATAAGAATATAAAATT--CACCACTTTATCTT 975

mouse_C ------------------------------------------------------------

* ** *** * * * * * *** * *** * *** * * **

BRNF

bovine_C CTTGGAGCTGGAAGTCTAAAAGCAGTGAAATCCCTTTTTTAATT-TAAAGGTTCTTGTC- 849

goat_C CTTGGAGCTGGAAGTCTAAAAGCAGTGAAATCCCTTTTTTCATT-TAAAGGTTCTTATC- 826

porcine_C TTAGGAGCTCGACATCTAAAAGTGATGAAATCTCTCTTTTTATTGTACATGTTCTTGCCA 979

human_C TTGTGAATGTGTGCTGTGAAG--AACTCCTTTACTGGGGTGATGGAACCAGTGGCTACAA 1033

mouse_C ------------------------------------------------------------

* ** * * ** * ** * ** * ** *

bovine_C -------TTCACAAC------------CCTTTCATATGATGACATTAATATTTG--AAGT 888

goat_C -------TTCCCAAT------------CCCTTCATACGATGACATAAATATCTGTGAAGT 867

porcine_C CCCCCT-CCCTCAACGAATTCTCCCCACCTTTTGTGTAATTATATTCATATCTGAGAGGC 1038

human_C AGTAAGGAGCTGGTTTACTGCTGTAAAGGGTTCGCGGCTTTGAATTTCAAGCTCTGGTTC 1093

mouse_C ------------------------------------------------------------

** * ** * *

bovine_C CCTCTTTTTTTTTTTTCCTTTTTTCTGCTTTGAAGTGA--CTAAATTTACATAACA---- 942

goat_C CCTCTTTTTCTTTTTTCCT--------CTTTGAAATGA--CTAAGTACACATAACA---- 913

porcine_C TCTATTTTTCATTTTCTTT--------TTCTGATATCA--TTAAACATACATAATAATAA 1088

human_C TGTGTCCTTGGGCACCTGCGCGTGAATCGTTGCCGCGAGGCTGGGCCAAGTTAAGGCCCC 1153

mouse_C ------------------------------------------------------------

* * ** ** * * * ***

OCT1 SORY

bovine_C --TTCATTT--CAATCATTTTAAGGATACAATTCAGTAGCTTCAGTTCAGTTCAGTCCCT 998

goat_C --TCCATTT--CAATCATTTTAAGGATAGAATTCAGTAGCT------------------- 950

porcine_C TATTCATTC--CATTCATTTTAAAGAT--------------------------------- 1113

human_C ACGCAGTTTGGCTTCCGGGCCAAGGAAGCCCCACAGGGTGCCCCACAGGGTGAAGCCCCA 1213

mouse_C ------------------------------------------------------------

** * * ** **

Region D

CLUSTAL 2.0.3 multiple sequence alignment

bovine_D TCAGTACATTCA-AATGTTGTACAATCAACAATTGTATGTAGCTCCGTAATATTTCACTG 59

goat_D TAGGTACATTCA-AATGTTGTGCAATCAACAATTGCATGTAGCTCCAGAATATTTCACTG 59

human_D ------------------------------------------------------------

porcine_D ------CATTCACAATGTTGTGCACCCATCATCTGTGTCTAGTTCCAGAATGTT-CATCA 53

mouse_D ------------------------------------------------------------

****** *********** ** ** ** * *** *** *** ** **

bovine_D ACCTAGAAAGAAAAGCTGAACAGCTTAGCAGTTACATTCCATGT--TCCTTCCCTCTTAT 117

goat_D ATCTAGAAAGAAAACCTGAACAGCTTAGCAGTTATTTCCCATGC--TCCTTCCCTCTTAT 117

human_D ----------------------------------------------------------AT 2

porcine_D CCCCAGGAAGACACCCTGCACCTCTTGGCAATTACCCCCCCTGCCCTCCTTCCCTCTTAT 113

mouse_D ------------------------------------------------------------

* ** **** * *** ** *** *** *** ** ** **************

bovine_D TCATTCCTGGGTAACCATCACTAATTTGGTTCTTTTCTCT---TAATTTGCCCTTTCTGG 174

goat_D TCATTCCTGGGCAGCCATCACTAACTTGGTTCTGTTCTCT---GCATTTGCCTTCTCTGG 174

human_D CCAGTCCTTAGCAACCAT---TAATCTGGTTTTAGTCTCTA-TTCATTTGCCTTTCCTGG 58

porcine_D TCAGTCCTCGGCAACCATCACAGATCTGGTCCCTGACTCTTATGTATTTGCTTCTTCTGT 173

mouse_D ------------------------------------------------------------

** **** * * ******* * **** **** ****** ***

bovine_D GGACT---TCATGTGAAGATAATCTTCCAGTAT-GTGGCCTCTTGTGTACC--------- 221

goat_D GGACT---TCACTTGAACATAATCTTCCAGTATTGTGGCCTCTTGTCTGCCATC------ 225

human_D ATATT---TCATATACATGGGATCATTCAGTAT-CTGGCCTCTTGTATCTGA-------- 106

porcine_D AAATGAAATCATTCAGTATATGGCCTCTGGTGT-CTGCCCTTTTTTTTCACATAAGGTGA 232

mouse_D ------------------------------------------------------------

*** * ** * ** *** ** * * *

MOKF

MOKF: Mouse Krueppel like factors

MOK2.01/02 Ribonucleoprotein

associated zinc finger

protein MOK-2

HOMF: Homeodomain transcription factors

DLX1/2/5, Distal-less 3, EN1 homeobox protein engrailed, HHEX, MSX1/2, NOBOX, S8.

NKXH: NKX homeodomain factprs

Hmx2/Nkx5-2 homeodomain transcription factor, NKX31 prostate-specific homeodomain protein, TTF1 thyroid transcription factor

BRNF: Brn POU domain factors

BRN2/3/4/5

RBIT: Regulator of B-Cell IgH transcription

Bright, B cell regulator of IgH transcription

OCT1: Octamer binding protein

OCT1/2/3

SATB: Special AT-rich sequence binding

Protein SATB1

FKHD: Fork head domain factors

FHXA/B, FKHRL1 (FOXO), FREAC2/3/4/7 fork head related activators (FOXF2, FOXC1, FOXD1, FOXL1), HFH1/2/3/8 (FOXQ1, FOXD3, FOXI1, Freac-6. FXF1), HNF3B (FOXA2), IlF1 (FOXK2), XFD1/2/3.

CREB: Camp-responsive element binding

Proteins

ATF, ATF2/6 activation transcription factors, c-Jun/ATF@ heterodimers, CREB, CREB1/2, CREB2CJUN, E4BP4, TAX/CREB complex, XBP1 X-box-binding protein.

PARF: PAR/bZIP family

DBP Albumin D-box binding protein, HLF hepatic leukemia factor, TEF Thyrotrophic embryonic factor, VBP PAR-type chicken vitellogenin promoter binding protein.

MYT1: MYT1 C2HC zinc finger protein

MyT1 myelin transcription factor, and MyT1L.

PLZF: C2H2 zinc finger protein

PLZF promyelocytic leukemia zink finger (TF with 9 Krueppel-like zink fingers)

MEF2: Myocyte-specific enhancer binding

Factor

MEF2, RSRFC4 related to serum response factor, SL1 member of RSRF

bovine_D TTTTTTAACACAGT---ATGATGTTTTCAAGGTTCCTCCACATTGCTGAATGTATCACTT 278

goat_D CTTTTTTGCACAGA---ATGATGTTTTCAAGGTTCCTCTACATTGCTGAATGTATCACTT 282

human_D CTTTTTCACACAGT---GTAACATTTTCAAGGTTCACCTATGTGGTGCCTTGTGTCATTT 163

porcine_D TGTTTTCACATAAGGTGATGATGTTTTCACAGTGCTTCCATAGTCCAGAATGTGTCACTT 292

mouse_D ------------------------------------------------------------

**** ** * * * ****** ** * * * ** *** **

HOMF NKXH RBIT BRNF OCT1 SATB/BRNF

bovine_D TTTGTC-TTTTAATTTCAA-CATTGAAAAGGTATGTATTT--TT-CTCATATATTAAACA 333

goat_D TTTGTC-TTTTAATTTCAA-CTTTGAGAAGATATGTATTT--TTTCTCATATATTAAACA 338

human_D TTTGCTATTTTCATGTCAGGTTTTGAAATGGTATGT--TT--TTTCTCATCTTGTAAGGG 219

porcine_D CTTTGCCTTTTAATTTCAA-ATTTGAAATGATATATATTTACTTTTTTTCTCATATATTA 351

mouse_D ------------------------------------------------------------

** **** ** *** **** * * *** * ** ** * *

FKHD NKXH

bovine_D ATTTCTAGGTTTATAAAATCTGCTTTTGTGTATGAGTGTCATTTAAACATTTGCTTCCAC 393

goat_D ATTTCTAGGTTTATAAAATCTGCTTTTGTGTATGAGTGCCATTTAAACATTTACTTCCAC 398

human_D ATTTCCTGTTGTAGAAAATCTGTTTCTGTGTATGACTGCAATTTAACCATTTTATTTGCT 279

porcine_D ATTTCTAGCTTTACAAAATCTGCTTTTATGTATGAGCGCAATTTAAACACTTGAGTCTGT 411

mouse_D ------------------------------------------------------------

***** * * ** ******** ** * ******* * ******* ** ** *

CREB PARF

bovine_D AGTAAGAGGCATGTTTTAAATTGCATGAAGTA-ATGCTATTTTCACATTTTA-------- 444

goat_D AGTAAGATGCATGTTTTTAATTGCATCAAGTA-ATAATGTTTTCACATTTTG-------- 449

human_D ATCCAGATACATGTTTTAACTTGAGCCAACTATATTTCCACTTTGTATAATGAATACTAA 339

porcine_D ACCAATACAGCTGCTTTAAATTAGATCAAGTA-ACAGTA-TTTCATATTTTA-------- 461

mouse_D -----------------------------GTCTCTAACATTCACAAACTTGAG------G 25

* * * ** *** * ** ** * *

MYT1

bovine_D --TTTGGATCTTTAATAGTTAACTTTA----ATGACTAAGATACAACTTA--CAGGATTG 496

goat_D --TTTGGATCTTTAATACTTAATTTTA----ATGAGTAAGATACAACTTA--CAAGACTG 501

human_D ATTTTGTATCTTAGATACTAAACTTTAGTAGATAACTAGGATACAAATTA--CAGTTTTC 397

porcine_D --TTTGAGTCT-GAACACTAAACTTCTTTGGGTGACTAAGATACAA-TTA--TGGAATTT 515

mouse_D GGTTTTGCTTGACAGTATCTAGGTTCATGAGATGGCTTCAAAGTAGATTAGTTGGGCTGG 85

*** * * * ** * * * * ***

bovine_D A-CACAT-----TTGGCTGAATAAAGTT--AGCCT--TTGAAGTTTCTACTATCATTGCA 546

goat_D A-CACAT-----TTGACTGAATAAAGTT--AGCCT--TTGAAGTTTCTACTGTCATTGTA 551

human_D A-TATTT-----TTCTTTTCATGGAATATAATTTT--TTAAAGTTTCTGATATTAATTCA 449

porcine_D A-TACATGCTTTTTGAATGAATAAAGTTAATTTTTGTTTGGACTTTTTAATATTATTGTA 574

mouse_D ACTAGGGAGGTCCTGAAGGAGGAGGGATAAATATT--------TTCTTACACACGTTAAA 137

* * * * ** * * *

FKHD BRNF OCT1

bovine_D TCATAAGAC--ATTGCTTAAATCA----TTTTTT---ATGCAGATCATTTTTATGTAGGA 597

goat_D TCACAAGAC--ATTGCTTAAATCA----TATTTTTA-ATGCAGATCATATTTATGTAGCA 604

human_D TTATAACATTTACTGGACAAACCAACCACATTTGGTTCTATGAACTCTTTTCATATTTTA 509

porcine_D CCATAACACTTATTGCTTGAACAT----CATTTGGTGATGCAGATTATTTGTATGTGATG 630

mouse_D TATTAAAATC-TTTGTTACAAGCA----CATTTTG----GTCAGTGGCTTTTAGCTCTTA 188

** * ** ** *** * * *

PARF

bovine_D CAAGCATTATTCAAATTATATTTTCTTAGGAATTTTGGCATCACTTTTATGAAATCTTGG 657

goat_D CAAGCATTATTCAAATTATATTTTATTAGGGGTTTTAGCATCACTTTTATGAAATCTTGG 664

human_D CGGG----ATTTTTATTTTGTTGTCTTAGGAATTGTGGCATCATTC------AGTTTTGC 559

porcine_D CA-------TTTAAATTTTATTTTCTCATGCATTGTGGCATCATTCTTATGCAGTCTTGG 683

mouse_D CAC------TTTAAGTTTTGACTTCTGTATCTGTCTGTCTCTGTCTGT-----CTCTGTC 237

* ** ** * * * * * * * *

MEF2 PLZF

bovine_D TAAGACTTT-TTATTATTTATATGTTATGTGAAATATATTTCATACAATATACAGTTGTT 716

goat_D TGAGACATT-TT-TATTTTTCATGTTATGCAAAATATATTTCATACAATATACAGTTGTT 722

human_D TGAGAGTTTATTTTTATTAATAGGTTCTGTGTACTATG-------CAACCTGTAGTTGTT 612

porcine_D TGAGACTTATTTTTACTTTTCATAGGTTATGTTCTGTG-------CAATCTACAGTCATT 736

mouse_D TGTCTGTCTGTCTTTGTCTGTCTGTCTGTTTCTCTTTG-------CATCACATCTCTGTC 290

* * * * * * ** *

SRFF CDXF NFKB

SRFF: Serum response element binding factors

CDXF: Vertebrate caudal related homeodomain

Protein

CDX1/2 Intestine specific homeodomain factor and mammalian caudal related intestinal TF.

NFKB: Nuclear factor kappa B/c-rel

NF-kappaB (p50 and p65), HIVEP1; ZAS Domain TF human immunodeficiency virus type 1 enhancer-binding protein-1 (HIVEP1), major histocompatibility complex-binding protein-1 (MBP-1), positive regulatory domain II-binding factor (PRDII-BF1)

HOXF: Factors with moderate activity to

homeodomain consensus sequence

Barx2, CRX, GSC, Gsh-1/2, HOX1, HOXA9, HOXB9, HOXC13, NANOG, OTX2, PCE1, PHOX2a/2b, PTX1 pituitary homeobox.

CLOX: CLOX and CLOX homology (CDP) factors

CDP cut-like homeodomain protein, transcriptional repressor CDP, CDPCR3, CDPCR3HD, CLOX, CUT2.

HNF1: Hepatic Nuclear Factor

HNF1.01/02/03

SORY: SOX/SRY-sex/testis determining and

related HMG box factors

HBP1, HMGA1/2, HMGIY, SOX5/9, SRY.

bovine_D ACTTCCATTTTTCAGATATGATTTTAAAACTT----GTATAGGAGAAGTCTCCTCCTATT 772

goat_D ACTCCCATTTTCCAGATATGGTTTTAAAACTT----TTATAGGGGAAGTCTCCTCATATT 778

human_D ACTCCTGTTTTGCAAATATGGCATTAAAACATTGGTGAGTGGGGGGACTTTCCTCCCATT 672

porcine_D ACTCCCATTTCCCAAACATGGTTTTAAAACT-------GTACGGGGACATTCCTCCTATT 789

mouse_D TTTTTGGTT---------------------------------------------------- 299

* *** ** * *** ******* * * * ***** ***

CDXF HOXF

bovine_D TTATTACA-TGAAAATACTCTATGTCAACTTTCAAGTTTGCCTTATGGATTTATTTGGAA 831

goat_D TTATTACA-TGAAAATACTCTGTGTCAACTTTCAAGTTTGCCTCACAGATTTATTTGGAA 837

human_D TTATTTCA-GCAAAGAAATCT-TGTCA--TTTCTCTTCAGACTTTAAGATTTATTGAGGA 728

porcine_D TTAGCACAATGTAAGTATTTTATGACAACTTCCAAGTCTGCCTTAACAATCTATTTGGAG 849

mouse_D ------------------------------------------------------------

*** ** ** * * * ** ** ** * * ** ** **** *

bovine_D GAAGGCTTCACTTATTTCATTTTGTAAAGCCTTACTTTTCAATTTTC---------TCCA 882

goat_D GAGGGCTCCAGTTATTTCATTTTGTAAAGCCTTACTTTTCAATTTT----------TCCT 887

human_D GAGTTATTCACTTGTTTAGTCTGGTAAACTGTGACCTTTAAATTTT----------GAAG 778

porcine_D GAAGTATTCACTTATTTCATTTGGTAAGCCATGACCTTTAAATTTTAAAAATGTTGTCCT 909

mouse_D ------------------------------------------------------------

** * ** ** *** * * **** * ** *** ******

BRNF CLOX

bovine_D CAGTTTGAACTACAATATAAAACA------ATTGATGGTTACAATTTAGAAATTTAGATT 936

goat_D GAGTTTGAACTATATTATANAANA------ATTGATGGTTACAATTTANAATTTTA---- 937

human_D GAGGTTGAACTAAAAGGTGAAATA--------TGATA-----AATTGATGGCTCTA---- 821

porcine_D AAGATTGAACTAGAATAAAAAAAATGATGAATCAGTGGCTGTAATTTAAAAAATTAGATT 969

mouse_D ------------------------------------------------------------

* ******** ** * * **** **

CREB PARF

bovine_D TATAAAGCTTTTTTTTTTGTTTTGTTTTATTTAGTTTTGATCATCTGTTAGGTAAAAGTG 996

goat_D --TACAGCATCATTGT---TTTAGTTTTGTTTAGTTTTAATCATCTGTTAGGTAAAAGTG 992

human_D --------TTTGTTGT----------GAATGGAAATTTAATT----------TAGACTTG 853

porcine_D T-TGTAAAAGCATTGTTCTTTTCATTTTGTTCTGTTTTGTACATTAGTGATGTAAAAGTG 1028

mouse_D ------------------------------------------------------------

*** *** ** * *** * **

bovine_D CAGAAGAGAGTGATGGATATTACTTCTGCAC-AGCCTACGCATCTAAGACACCACACACC 1055

goat_D CAGAAGAGAGTGATGGATATTACTTCTGCACCAGCCTACCCATCTAACATACCACACACC 1052

human_D TAAAAGATTGTTTTTTAAATTGCT-------------AATTGTGTGACAGTGAAGAGAAT 900

porcine_D AAGAAGACAGAGGCCAGTGGTGAGCATACTC----CTGACAGTCTCGAGAACAAAATGGT 1084

mouse_D ------------------------------------------------------------

* **** * * * * * *

bovine_D AACCCCC-----ACCCCCAC------------------CCTCCCACTTTTCCTACTCCCC 1092

goat_D AACCCCCCTTTCACCTCCGCACCCAGTCCCCCGCCCAGCCTCACACTTTCCCTACTCCCC 1112

human_D GACTTCA------------AA-------------------------ATATCCCACTATAC 923

porcine_D CCCTTTGAAGTGTTATGTTCACCTCGGGAAACTTTGAGTTCCAAGGTTATCTGTTTTTCT 1144

mouse_D ------------------------------------------------------------

* * * *

bovine_D AACCGTCAGAACAAAAAAATAAAAATCCAGTCCCTTTGAAGTGTTATATTAACACCAGG- 1151

goat_D CACCCTCAAAACAAACAAACAAAA-CCCAGTCCCTTTGAATTGTTATTTCAATACCAGG- 1170

human_D AATCTGCAAAG-AAAAGAATTGTGTCCC---CTTTTTAGTGTAGCTTAACACTTCACTG- 978

porcine_D CTTAATGGAACCATTTTAAGTCTCTTGCCAGAATTCAGCCTCTGTCTGCCAGTGCCGAAT 1204

mouse_D ------------------------------------------------------------

* * ** * * * *

bovine_D -GAACTGCTT-GGGTACCAAGGTTATGTGTTTTTT-CTTTTAATGGAACAGGTTTTT--- 1205

goat_D -AAACTGCTT-GGGTGCCAAGATTAAGTGTTTTTTTCTTTTAATGGAACAGTTTTTA--- 1225

human_D -AAACTGTTTTGAGTTCTTAGGTCATATTTTTTTTTCTCTAAACGAAACAATTACTTTTC 1037

porcine_D TGAAAGAATTGAAACGCAGAGATGGGGTTTGGGGTAAAGAAAAAAAAAATAGCTTTAT-- 1262

mouse_D ------------------------------------------------------------

** ** * ** * * * ** **

OCT1

bovine_D ----AATCTAATTTTAGTTGTATCTGAGATTGCCTGTTAAATATGTGTTAGTATATATTA 1261

goat_D ----TGTAAAATTTTAGTTGTATCTGAGATTGACCATTAAATATGTATTAGTATATATTA 1281

human_D TAAAAGTCAAATGTTAGCCATCCTAGAAGTTGGGCATAAAATACTTGTAAGTATATGCTA 1097

porcine_D TGCTTTGCCAGGCAAAGGAGGATCAGAGCAGGCTAATTA---ATGCCCTAAAGACTGTGA 1319

mouse_D ------------------------------------------------------------

* ** ** * * * * * *

bovine_D GCATTCTGAAAGTCGTTAGCACAGATAATAGAATTACCAGTTATTAGCTACTGGAATAC- 1320

goat_D GCATTCTGAAAGTCGTCAGCTTATATC---CAGT--CCAGTTATTAACTACTGGAATAT- 1335

human_D ATATTCTGA----------------------------TACTTAATGCCTG-TGAAAAAT- 1127

porcine_D AGACTGTGCTCCCCCACGCCCCACCCCC-----CCCCCAAAGAATTGTGAGGAGTTTACA 1374

mouse_D ------------------------------------------------------------

* * ** * ***

HNF1 OCT1

bovine_D GTACATAGATTTTTCAGCTGCACTTTGAGCCTAAGTGGAGAAGCAGGGTTAGTGATTAGC 1380

goat_D GTACATAAAATTTTCAGCTGTACTTCAAGCCTAAGTGGAGAAGCGGGGATAGTGATTAGC 1395

human_D GTGTATAGAATTTTCAATT----TTTAAATAGAAGTGAAGAAAAAGCGATAATAATTACT 1183

porcine_D GTAAAAAAGGAGAAAAACAGGTTTGCCGATAGAACCGGATTGGGACACACATGCATTCTT 1434

mouse_D ------------------------------------------------------------

** * * * * ** * * ***

bovine_D ATGGATTGGGTATCC---TGTGTATTCAAATATATAACAGGATTGGGC-TGTTTTTCATC 1436

goat_D ATGGATTTGGTATCC---TGTGTATTCAACTATATAACAGGATCGGGC-TGTTTTTCATC 1451

human_D ATAAATTCAATATGCAGTTATGTATGTATGTGTGTGGTTAAGACAATT-AGGTTCTCATT 1242

porcine_D CTTTCTTTGGGGGAAT-CTTTGTCCTCAAAACTGAGTCAGAGATGTTCATGATGGTGGTC 1493

mouse_D ------------------------------------------------------------

* ** * *** * * * * *

bovine_D TTTTTTTGTTT-----AAGATAACA-TTCTCTTA---------CATTCAT-AACCGT--A 1478

goat_D TTTTTTTTTTTTTTTTAATATAACA-TTCTCTTA---------CATTCAT-AACCAT--A 1498

human_D AAGCTTTGTTTTTTTAAAGATAACA-TACACATA---------TATTGAT-AATGAT--A 1289

porcine_D TTCTGGGTTATTGCCTAGGATAACAGTGCTTGCAAAAAGGGCGTACTGATCAGAGATTAG 1553

mouse_D ------------------------------------------------------------

** * ****** * * * * * ** * *

SORY

bovine_D GACAATTTGCTAGTATGTCTGCTGCACCTTCATCCTTTGAAATAAGTATATGA-AAATAA 1537

goat_D AACAATTTGTTAGTATGTCTGCTGAACCTTCATCCTTTGAAATAAGTATATGA-AAATAA 1557

human_D AACAATTCATATAGCTTTTTG-TGTCCTCTCGTTTTGTGACATAAAAGGTCAATGAAAAA 1348

porcine_D AACAAACCAGGAAAGTTCCTGACAAACATCAGGTACTAGTCATCTTTAACCCACAGGCCA 1613

mouse_D ------------------------------------------------------------

**** * ** * ** * *

bovine_D CT-TCATAATGACACTT--TTTGTATTTTTAAGCA-GGTGTTAGCACATTCACAAATTCT 1593

goat_D CT-TCACAATGACACCT--TTTGTATTTTTAAGCA-GGTATTAGCGCCTTCACAAATTCT 1613

human_D AT-TGGCGATTAAGTCAAATTCGCATTTTTCAGGACAGCAGTAGAGCAGTCAGGGAGGCA 1407

porcine_D TTATGCTCAGAGGGCCTGACCTTTAGCTTAGAGGT-GGTCCTGTTTAGGGTGCAATTAAG 1672

mouse_D ------------------------------------------------------------

* * * * ** ** *

bovine_D GATTAGATGTAAACAAAGAAGAAAGCAGAGCC--TTAATATCCTGTTAAGTAGCTTT--- 1648

goat_D GATTAGATGTAAACAAAGAAGAAAGCAGAGCG--TTAATATCCTGTTAAGCACCTTT--- 1668

human_D GATCAG-------CAGGGCAAGTAGTCAA-CG--TTACTGAATTACCATGT---TTT--- 1451

porcine_D TCAGGGAGAAGGACAAGGAAGGACTCTAAGCTGTTTAACTTCAAAGTAGTCAATTTCTAA 1732

mouse_D ------------------------------------------------------------

** * * * * *** * **

bovine_D --GCTTGAGAAAGAGTAGGTTGATGGGTTT-----GGGCTGACTG-CCAGGACGTATTGA 1700

goat_D --GCTTGAGAAAGAGTACGTTGGAGGGTTT-----GGGCTGGCTG-CCAGGAGGTATTGA 1720

human_D --GCTTGAGAATGAATACATTGTCAGGGTACTAGGGGGTAGGCTGGTTGGGCGGGGTTGA 1509

porcine_D AAGACACATAAGGAATATAACTTTTCTCTCAGGAGTATGAGGCAACCTGCTACATATCTA 1792

mouse_D ------------------------------------------------------------

* * ** ** ** * * * *

bovine_D GGGGAGGTA---TTGGGGGCGGAGAAATAAATATTTCACTGTATATATTGCACTAA-GTC 1756

goat_D GGGGAGGTA---TTGGGGGCGGAGAAATAAATATTTCACTGCAAATTTTGCACTAA-GTC 1776

human_D GGG--GGTG---TTGAGGGCGGAGAAATGCAAGTTTCATTACAAAAGTTAACGTAA-CAA 1563

porcine_D ATGTTATTTGACTCTGAGACTGGCCATTAAATAGATGCTAGTATATATTAACATACTGAT 1852

mouse_D ------------------------------------------------------------

* * * * * * * * * * * ** *

bovine_D AGTCTGTGGTAAGAACA---ACTTATGAATAGCACCATAATTTTTAGAACGCTTACACCG 1813

goat_D AGTCTCTGGTAAGAACA---GCTTATGAATAG---------------------------- 1805

human_D AGAATCTGGTAGAAGTG---AGTTTTGGATAGTAAAATAAGTTTC-GAACTCTGGCACCT 1619

porcine_D AATCATCAGCAGAGGTATTCACATTTAGAATTCCCAATTATTTGC-CCACAGGAAAACTT 1911

mouse_D ------------------------------------------------------------

* * * * * *

bovine_D CATATTACTTCCTCCCCTTTT--------------------------------------- 1834

goat_D ------------------------------------------------------------

human_D TTCAATTTTGTCGCACTCTCCTTGTTTT-------------------------------- 1647

porcine_D AACAGAATTTTCAATAGTTCTTTAAGTAGAAGTGAAGAAANAAGAATGACGACTAGCATT 1971

mouse_D ------------------------------------------------------------
